# Analysis of Post-Colonoscopy Colorectal Cancer and Its Subtypes in a Screening Programme

**DOI:** 10.3390/cancers13205105

**Published:** 2021-10-12

**Authors:** Saloa Unanue-Arza, Isabel Idigoras-Rubio, Maria Jose Fernández-Landa, Isabel Bilbao-Iturribarria, Luis Bujanda, Isabel Portillo

**Affiliations:** 1 Department of Nursing I, Faculty of Medicine and Nursing, University of the Basque Country (UPV/EHU), 48940 Leioa, Spain; 2 Biocruces Health Research Institute, Clinical Nursing and Community Health, 48903 Barakaldo, Spain; 3 Osakidetza Basque Health Service, Basque Country Colorectal Screening Programme, 48011 Bilbao, Spain; isabel.idigorasrubio@osakidetza.eus (I.I.-R.); MARIAISABEL.BILBAOITURRIBARRIA@osakidetza.eus (I.B.-I.); MARIAISABEL.PORTILLOVILLARES@osakidetza.eus (I.P.); 4 Biocruces Health Research Institute, Cancer Biomarker Area, 48903 Barakaldo, Spain; 5 Osakidetza Basque Health Service, OSI Bilbao-Basurto, Rekalde Ambulatory, 48002 Bilbao, Spain; mariajose.fernandezlanda@osakidetza.eus; 6 Biodonostia Health Research Institute, 20014 San Sebastian, Spain; LUIS.BUJANDAFERNANDEZDEPIEROLA@osakidetza.eus; 7 Center for Biomedical Research Network for Liver and Digestive Diseases (CIBERehd), 20014 San Sebastian, Spain; 8 Department of Gastroenterology, Donostia University Hospital, Faculty of Medicine and Nursing, University of the Basque Country (UPV/EHU), 20014 San Sebastian, Spain

**Keywords:** colorectal neoplasms, early detection of cancer, colonoscopy, interval cancer, adverse effects, risk factors

## Abstract

**Simple Summary:**

This study responds to the algorithm proposed by the World Endoscopy Organization (WEO) and adapts well to the surveillance of the adverse effects of the population-based colorectal cancer (CRC) screening programmes (interval cancers). In our case, the application of the aforementioned algorithm to the Basque Country Programme, which began in 2009 and reached coverage of around 100% in 2014, has been an opportunity to evaluate in a standardised way the cancers detected after a colonoscopy carried out following a positive Faecal Immunochemical Test (FIT). All the characteristics found in these cancers and their relationship with the index colonoscopy have been described. The differences in both the stage and the quality of the colonoscopies and the lesions detected must be taken into consideration, in order to evaluate the surveillance and to reduce one of the adverse effects on a CRC screening programme, as far as possible.

**Abstract:**

Using the algorithm of the World Endoscopy Organisation (WEO), we have studied retrospectively all colorectal cancers, both detected and non-detected by the Basque Country screening programme from 2009 to 2017. In the screening programme 61,335 colonoscopies were performed following a positive Faecal Immunochemical test (FIT) (≥20 µg Hb/g faeces) and the 128 cases of post-colonoscopy colorectal cancer (PCCRC) detected were analysed. Among them, 50 interval type PCCRCs were diagnosed (before the recommended surveillance), 0.8 cases per 1000 colonoscopies performed, and 78 non-interval type PCCRCs (in the surveillance carried out at the recommended time or delayed), 1.3 per 1000 colonoscopies. Among the non-interval type PCCRCs, 61 cases were detected in the surveillance carried out at the recommended time (type A) and 17 when the surveillance was delayed (type B), 1 case per 1000 colonoscopies performed and 0.28 cases per 1000 colonoscopies performed, respectively. Interval type PCCRC is less frequent than non-interval type PCCRC. In interval type PCCRCs, CRCs detected in advanced stages (stages III–IV) were significantly more frequent than those detected in early stages, compared to those of non-interval type PCCRCs (OR = 3.057; 95% CI, 1.410–6.625; *p* < 0.005). Non-interval type B PCCRCs are less frequent than non-interval type A PCCRCs, but the frequency of advanced stages is higher in interval type B PCCRCs.

## 1. Introduction

The Colorectal Cancer (CRC) Screening Programmes have the objective of reducing the incidence and mortality linked to this cause. The screening test chosen by the majority of European Union (EU) countries is the Faecal Immunochemical Test (FIT).

Although the FIT has proven its effectiveness [1,2], and colonoscopy is the Gold standard for the detection of CRC and premalignant lesions [3], not all CRCs of the population participating in the screening are detected within the prevention programme and are thus considered false negatives. This is one of the adverse effects of screening programmes, referred to as Interval Cancer (IC). According to the definitions proposed by the group of experts of the World Endoscopy Organization (WEO), there are two specific entities that have historically been categorised as CRC not detected by the programmes: (a) FIT Interval Cancers (FIT-IC), defined as CRCs diagnosed between two invitations, after a negative FIT result and before the next invitation; (b) post-colonoscopy colorectal cancers (PCCRC). These are divided into interval type PCCRCs, where the cancer is identified before the next recommended surveillance examination, and non-interval type PCCRCs, where CRC is diagnosed at or after the recommended surveillance interval. Likewise, another subdivision analyses non-interval type PCCRCs according to the moment of diagnosis, type A (detected at recommended surveillance interval), type B (detected after recommended surveillance interval) and type C (detected when no surveillance interval has been set) [4].

The study of these entities allows us to evaluate both the diagnostic capacity of the screening test, as well as the quality of the colonoscopies carried out after both a positive result in the FIT and in the surveillance.

For this reason, and following international recommendations, it is essential to count on a consensus of definitions and indicators, which will allow us to evaluate and compare results with other programmes [5,6,7]. In order to do so, we have taken into account the recommendations of the European Guidelines for Quality Assurance in Colorectal Cancer Screening and Diagnosis [8,9], as well as those adopted by The Spanish Cancer Screening Network [10]. The endoscopic findings of the screening colonoscopy have these surveillance recommendations: (1) Normal or Hyperplasic Polyps-invitation to CRC screening programme with FIT in 10 years; (2) Low Risk: 1 or 2 adenomas <10 mm and without a villous component—invitation to CRC screening programme with FIT in 5 years; (3) Intermediate Risk: 3 to 5 adenomas and/or 10 to 19 mm and/or villous component—surveillance colonoscopy in 3 years; (4) High Risk: ≥5 adenomas and/or ≥20 mm—surveillance colonoscopy recommended within one year. In 2014, new definitions were agreed upon by the Expert Working Group on interval CRC of the Colorectal Cancer Screening Committee of the World Endoscopy Organization (WEO), which we have taken into consideration in our study, in order to classify the detected and non-detected lesions by the Basque Country programme, following the strategy aimed at improving its effectiveness.

The assessment of the quality criteria for the colonoscopy [11], such as the adenoma detection rate by the endoscopist, caecal intubation rate and quality of colonic cleansing by endoscopist are necessary parameters in order to measure, assess and possibly predict the rate of PCCRC and above all, represents an important quality indicator [12,13,14,15]. The detailed study of each case will enable the implementation of correction measures in screening and surveillance colonoscopies. Moreover, taking into account the sociodemographic, comorbidity and deprivation index [16,17] will allow the adjustment of results for future intervention in the screening and surveillance.

This study is the continuation of the one published before [18], including a wider period of surveillance, allowing us to use the proposed classification by the WEO in the case of PCCRCs.

The main objective is to evaluate the CRC cases detected by the Basque Country programme between January 2009 and December 2017, specifically those of the Post-Colonoscopy Colorectal Cancers in order to know about their common and differential characteristics so as to improve the CRC screening process and surveillance.

## 2. Materials and Methods

### 2.1. Study Design and Study Population

A retrospective observational study of CRCs detected through the Basque Country screening programme since the programme started, from 1 January 2009 to 31 December 2017 in individuals born between 1 January 1940 and 31 December 1967, was carried out. Women and men of 50 to 69 years old, living in the Basque Country (one of the 17 autonomous regions in Spain) and subject to screening (611,000 people) were invited by means of a biennial quantitative faecal immunochemical test (FIT) with a threshold ≥20 μg Hb/g OC-Sensor^®^ Micro (Eiken Chemical Co., Ltd., Tokyo, Japan).

### 2.2. Data Sources

All detected and non-detected cases by the CRC screening programme are monitored following by the coordination centre following the Clinical Practice Guidelines. We considered CRC the codes 1530–1548 of the International Classification of Illnesses-9 [19] in primary and secondary diagnosis IC-DO-10 (C18–C20) [20]. All cases ≥pT1 were considered CRC, coded according to the criteria of the American Joint Committee on Cancer (AJCC) [21].

The following sources were used for the study:

- Database of the Basque country’s CRC screening programme;
- Basque Country Mortality Registry (completed up to 2020);
- Basque Country Cancer Registry (completed up to 2017);
- Discharges from the Standardised Virtual Medical Registries (Osabide) of the Basque Health System—Osakidetza (completed up to 2020).

Selection of cases:

- Screen-detected CRC: CRCs detected within the screening programme after a positive FIT;
- FIT-IC: CRCs diagnosed between two invitations, after a negative FIT result and before the next invitation and followed 2 years after the last negative test (70–71-years-old);
- FIT positive without colonoscopy performed after six months and CRC detected;
- Post-imaging CRC: CRCs detected after an imaging procedure and reported as normal;
- Interval Type PCCRC: CRCs detected before the next recommended surveillance interval;
- Non-interval type PCCRC: CRCs detected at or after recommended surveillance interval:
  - Non-interval type A PCCRC: CRCs detected at recommended surveillance interval;
  - Non-interval type B PCCRC: CRCs detected after recommended surveillance interval;
  - Non-interval type C PCCRC: CRCs detected when no surveillance interval has been set.

In all cases, colonoscopies were analysed according to the quality criteria proposed by the Clinical Practice Guidelines for quality in colonoscopy screening and colorectal cancer of the AEG-SEED: caecal intubation, colonic cleansing according to the Boston scale (adequate quality considered to be a score of ≥6 with a minimum score of 2 in each of the three sections of the colon), location and characteristics of the previous polypectomy (type of resection: piecemeal or bloc, grade of dysplasia and morphology of the resected adenoma in the screening colonoscopy according to the Paris classification). Moreover, the characteristics of the tumour were described, such as the stage and the degree of differentiation (according to the WEO Consensus Statements).

The deprivation index was included, assigned to each patient according to the census tract in five quintiles, from very low (high socioeconomic status) to high (the lowest socioeconomic status) [22].

### 2.3. Main Outcome Measures

The main result was the possibility of obtaining the characteristics of PCCRCs according to the classification proposed by the Interval Cancer Experts Group. The non-interval type C PCCRC was not studied since no cases without surveillance recommendation were registered.

### 2.4. Statistical Analysis

Continuous variables were described by mean and standard deviation. The categorical variables were described by frequencies and percentages. Comparisons between categorical variables were performed using the chi-square test or Fisher’s test when the expected frequencies were less than 5. The confidence intervals were calculated at a 95% confidence level, and all results were considered statistically significant for *p* < 0.05.

A logistical regression analysis was carried out with the significant variables in order to estimate the Odds Ratios with a confidence interval of 95%. Values *p* < 0.05 were considered statistically significant.

Non-parametric tests were carried out for the analysis of the time elapsed between the performance of the index colonoscopy and the diagnosis of CRC: Mann-Whitney U test for the contrast between the periods of interval type PCCRCs and non-interval type PCCRCs, and Kruskal-Wallis for the analysis of the differences between groups, in relation to the different findings in each of the PCCRCs (the latter depends on the importance of including the analysis of both interval and non-interval types).

**Figure 1:**
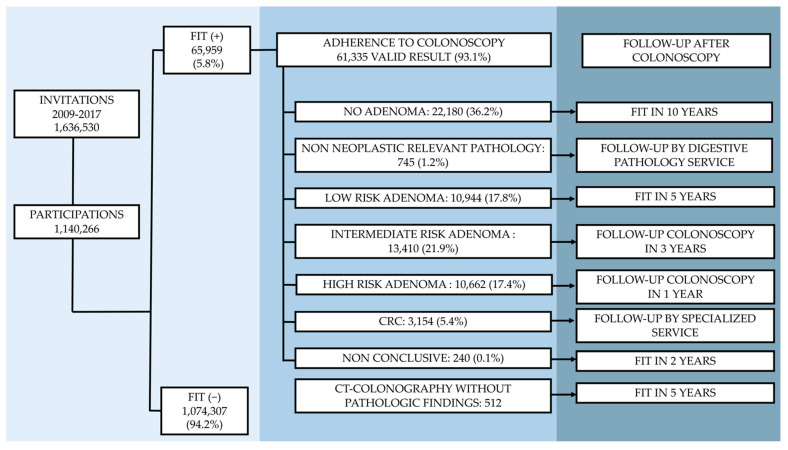
Findings in the colonoscopies carried out, and corresponding recommended surveillance according to the European Guideline for Quality Assurance in Colorectal Cancer Screening and Diagnosis and the consensus of the Spanish Cancer Screening Network [9,23].

The incidence is shown as the number of cases per 1000 person-years, along with the confidence interval at 95%. In order to do so, in each case we used the corresponding number of cases detected after the index colonoscopy (Figure 1), and the median of the surveillance period for each patient according to the group they belonged to, interval type PCCRC and non-interval type PCCRC (non-interval type A PCCRC, non-interval type B PCCRC).

Correlations between variables have not been considered. The analysis was carried out by a biomedical statistician using the statistical programs SPSS 23.0, IBM (Armonk, NY, USA) and R, version 4.0.1.

## 3. Results

### 3.1. Study Population

There were 1,636,530 invitations registered in the studied period. The overall participation rate was 69.7%, being higher in women than in men (73.76% vs. 68.86%; OR = 1.27; 95% CI, 1.236 to 1.280; *p* = 0.000). With regards to age, both in men and in women, participation was significantly higher in the group of 60–69-year-olds than in the group of 50–59-year-olds (*p* = 0.000).

The negative FIT cases (<20 μg Hb/g faeces) were 1,074,307 (94.2%). The positivity rate was higher in men than in women (7.3% vs. 4.4%; OR = 1.679; 95% CI, 1.670 to 1.724; *p* = 0.000). With regards to age, in men positivity was lower in the group of 50–59-year-olds than in the 60–69-year-old group. (6.1% vs. 8.8%; OR = 0.681; 95% CI, 0.667 to 0.696; *p* = 0.000). In women, positivity was lower in the 50–59-year-old group than in the 60–79-year-old group (3.9% vs. 6.4%; OR = 0.745; 95% CI, 0.727 to 0.764; *p* = 0.000). 93.1% underwent a screening colonoscopy (61,335) with a definitive diagnostic and surveillance guidelines according to the detected risk. Figure 1 shows the findings in the colonoscopies carried out and the recommended surveillance for each of them.

**Figure 2:**
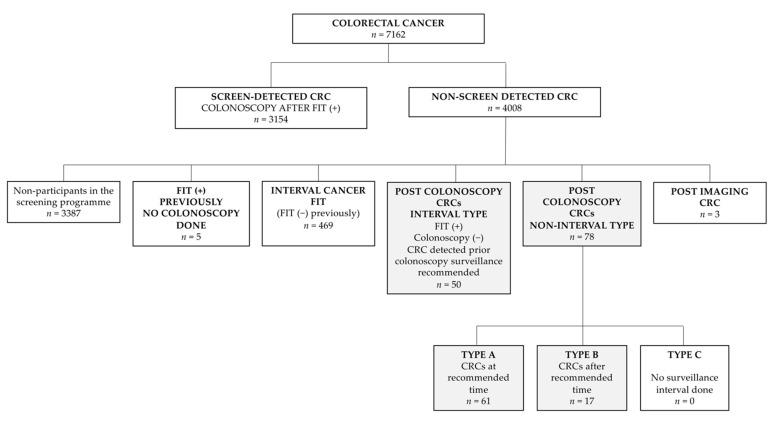
CRCs detected in the period 2009–2017 according to the group of experts in Interval CRC WEO [4]. The cancers that are the object of study of this paper have been shaded in.

Figure 2 shows all the CRCs detected in the analysed period according to the classification of the WEO group of experts are shown in Figure 2. The CRCs detected in the private health system (16 cases) were included for the total count, in addition to those specified in the figure, but excluded from the analysis.

Table 1 shows the incidence per 1000 person-years of all types of PCCRCs in general, and in relation to the index colonoscopy findings. The general incidence of interval type PCCRC in participants in the screening programme who undergo a colonoscopy is 0.116 cases/1000 person-years, for all the findings since the index colonoscopy, and 0.160/1000 person-years for the non-interval type PCCRC. In relation to high-risk adenomas, incidence of PCCRC is lower in the interval type PCCRCs than in non-interval type PCCRCs, with 0.027 cases/1000 person-years vs. 0.662 cases/1000 person-years respectively. However, when analysing non-interval type PCCRCs specifically, high-risk adenoma was the finding which showed the highest incidence (0.662 cases/1000 person-years).

### 3.2. Patient and Tumour Characteristics of PCCRC Cases

Table 2 shows the sociodemographic and tumour related characteristics, of the PCCRC divided into (a) Interval type PCCRC and (b) Non-interval type PCCRC. There were no statistically significant differences except for tumour stage, 54% of the interval type PCCRC and 78.2% of the non-interval type PCCRCs were found in an early stage (*p* = 0.004). Advanced stage PCCRCs are three times more likely to be interval type PCCRCs than non-interval type PCCRCs (OR = 3.057; 95% CI, 1.410 to 6.625; *p* = 0.05). In relation to the period of time elapsed (months) between the performance of the index colonoscopy and the diagnosis of CRC, we observed that, in the cases in which a low-risk adenoma or an intermediate-risk adenoma is detected, there are statistically significant differences. On the other hand, in relation to interval type PCCRCs, the cases in which a low-risk adenoma has been detected, the period of time elapsed until the detection of CRC is statistically significantly higher than when an intermediate-risk adenoma or a high-risk adenoma is detected (*p* = 0.009).

Table 3 describes the characteristics of colonoscopies in which PCCRC was later detected, both interval type PCCRC and non-interval type PCCRC, according to the colonoscopy quality criteria and lesions detected in the index colonoscopy. No statistically significant differences were found in terms of quality, but differences were found in terms of resection and findings detected in index colonoscopy. In the group of non-interval type PCCRC, 71.8% were classified as high-risk adenoma in the index colonoscopy. However, in the interval type PCCRC, 38% showed no adenomatous lesion and 36% had low-risk adenoma in the index colonoscopy. In terms of the type of polyp according to the Paris classification, 58.3% of the interval type PCCRCs were sessile (Is), whereas 39.6% of the non-interval type PCCRCs were pedunculated (Ip). No statistically significant differences were found in terms of location of the tumour. With regard to the location of the tumour in relation to the finding in index colonoscopy, it coincided in 76.0% of the interval type PCCRCs. In 32.1% of the non-interval type PCCRCs the tumour did not coincide with the location of the detected and resected lesion (OR = 6.713; 95% CI, 3.003 to 15.009; *p* = 0.000).

In Table 4 the comparative analysis between non-interval type A and type B PCCRCs is shown. No statistically significant differences were found in any of the variables related to the sociodemographic characteristics or the tumour characteristics. However, despite the delay in performing the colonoscopy of surveillance in the non-interval type B PCCRC cases as opposed to those performed following the recommendation and risk of non-interval type A PCCRC, no significant differences were found in the stage of the tumour, even though the percentage of CRCs in advanced stages was higher in the non-interval type B PCCRC. Regarding the characteristics and findings of the index colonoscopy, statistically significant differences were found in quality of colonic cleansing at index colonoscopy as shown in Table 5, in fact, poor colonic cleansing (lower than 6 in the Boston scale) was more frequent in non-interval type B PCCRCs in relation to non-interval type A PCCRCs (35.3% vs. 3.3%). Moreover, the percentage of high-risk adenoma was significantly higher in non-interval type A PCCRCs.

## 4. Discussion

In this study, both the detected and non-detected CRCs by the CRC screening programme were classified according to the WEO proposal, specifically analysing the interval and non-interval PCCRC types in the Basque Country between 2009 and 2017 [4]. This monitoring has been possible due to the availability of both cancer and hospital discharge registries, which despite their limitations provide us with a good monitoring tool for the adverse effects of screening programmes [24].

Like in the study carried out by Mlakar et al. in 2018 in Slovenia [25] our results did not show statistically significant differences either according to age, sex, location of the tumour or deprivation index among the two types of PCCRC detected. With regard to the location of the CRC, the results of our study differ from those of Dossa et al. due to the fact that although a higher frequency of CRC in distal colon was observed (38% and 37.2%) in both interval type and non-interval type PCCRCs in our study, they observed that the majority of cases were located in the proximal colon (54.2% of 367 PCCRCs) [26]. However, it should be noted that these studies included persons with symptoms with previous pathologies such as inflammatory bowel disease, hereditary syndromes, previous CRC, polyposis or diverticulosis. In the case of the Basque Country, these were not considered, as the participants in the screening programme were average risk, asymptomatic people of 50 to 69 years old. The recent article by Beaton et al. (2021) found that 48 of the 527 cases studied were PCCRCs, of which 7 (15%) were interval type PCCRCs and 41 (85%) non-interval type PCCRCs. In our study, they were 50 (39.1%) and 78 (60.9%) respectively, although it should be noted that in Beaton’s study, the percentage of the CRCs detected in screening programmes was 8% of all cases analysed [27]. Likewise, the importance that these authors give to the adherence to the surveillance in order to avoid the numerous cases of non-interval type PCCRC is ratified by the results of our study. The percentage of non-interval type PCCRCs (78/128 cases) was lower than that reported in their study due to the fact that the protocol of the screening programme in the Basque Country includes the recommendation of surveillance for all colonoscopies and compliance is monitored.

In our study PCCRCs were mostly detected in their early stages (I and II), in fact, the probability of detecting a tumour in an advanced stage in the interval type PCCRCs is three times higher than in non-interval type PCCRCs. Our results are similar to those of the study by Anderson et al. and Willington et al. in which 63.6% of the 107 PCCRCs analysed and 63% of 46 PCCRCs analysed were diagnosed in the early stages, respectively [28,29]. Regarding PCCRCs on the whole, however, the fact that in these studies there is no differentiation between interval type PCCRC and non-interval type PCCRC does not allow us to contrast our results following the proposed classification.

The interval type PCCRCs could correspond to new, rapidly evolving lesions or to undetected lesions in the colonoscopy screening, due to the fact that 76% were detected in a different location to the adenoma resected in the index colonoscopy. In studies published in this regard, the results differ from ours. On one hand, Robertson et al., in 2014, concluded that the undetected lesions in the colonoscopy were responsible for 50–60% of the interval cancers and Zhao et al., in 2019, carried out a meta-analysis in which they concluded a rate of 26% missed adenomas [30,31]. This would indicate that these lesions not detected in the index colonoscopy could progress towards an invasive cancer and be detected as interval or non-interval type PCCRC, depending on the date of diagnosis. On the other hand, Hsu et al., in 2021, observed that 68.8% of interval type PCCRCs developed from a new lesion [32]. Likewise, Jennings et al. observed that 47% of the interval type PCCRCs detected in a programme in which guaiac faecal occult blood test is used as the screening test were related to non-detected lesions, being 27% likely new CRCs and 20% were related to missed detection [33]. In our study, adenomas were removed in 31 of the colonoscopies that were later interval type PCCRCs (62%) and in 73 of the 78 CRCs classified as non-interval type PCCRCs (93.6%). Despite this, the programme registers parameters of quality in accordance with the recommendations of the ESGE [34], including caecal intubation, colonic cleansing and adenoma and CRC detection rate (23.9 and 3.4 respectively per 1000 participants) [35]. This could be related to the low percentage of interval type PCCRCs that have been identified in this study. The results are in accordance with those found by Nally et al., who, in their study published in 2019, found that a low adenoma detection rate was directly linked to the detection of interval cancers [36]. On the other hand, in the study by Farrar et al., in 2006, a relationship was found between incomplete polypectomy and the detection of interval cancer [37].

With regards to non-interval type PCCRCs, 71% of CRCs were found in the colonoscopy for the surveillance of high-risk adenomas detected in the index colonoscopy. The location of the CRC coincided with the previously resected lesion (67.9%) although 78.2% were found in an early stage. This would indicate the appropriateness of the surveillance of these cases being carried out within 1 year [13] while questioning the proposed surveillance after 3 years of the index colonoscopy, except in sessile lesions ≥20 mm resected in piecemeal (6 months) [38]. Even though these new recommendations will allow us to reduce the burden on healthcare of the follow-up colonoscopies, it will be essential to monitor all the cases thoroughly due to the fact that in our study 20 non-interval type PCCRCs would have been considered interval type.

On the other hand, differences were found with regards to the morphology of the lesions retrieved during index colonoscopy, 58.3% were sessile (Paris Is) in the interval type PCCRCs and 39.6% pedunculated (Paris Ip) in the non-interval type PCCRCs. Accordingly, Yamaguchi et al., in 2020, observed that the type of lesion and the retrieved lesions that had not been retrieved for pathological study were related to a higher risk of PCCRC [39]. This was not observed in our study due to the fact that the programme does not assign a risk until the complete resection of the lesions, be it in one or in several sessions.

Another result that should be highlighted in this study is the possibility of establishing and comparing the adherence to the surveillance colonoscopies of the non-interval type A and type B PCCRCs. Accordingly, 61 people with CRC diagnosis had it carried out in the recommended period and 17 at a later date. The quality of the colonic cleansing was 13.5 times worse in the non-interval type B PCCRC ones than in those of non-interval type A PCCRC. There were no statistically significant differences in the stage of the tumour. This would indicate that a delay in undergoing a surveillance colonoscopy does not have serious consequences, although a larger sample would be needed to confirm it. Moreover, the fact that all colonoscopies are subsequently followed up is a quality criterion that is based on the protocol of the programme, whereby the Endoscopy Unit carries out an informed recommendation once the colonoscopy and pathological anatomy reports have been assessed and it is filed in the clinical history.

Nevertheless, even though it is essential to keep monitoring all cases, due to the size of our sample and the surveillance period, the consensus proposal of the group of experts on interval cancer of the WEO [40] is of great value in order to compare the quality of the CRC screening programmes and to improve the processes and surveillance, taking the index colonoscopy as the starting point.

Moreover, a limitation of this study is that the sample is small once disaggregated into the different subtypes according to the classification proposed by the experts in IC. It would be very interesting to continue studying the cases over a length of time in order to increase the sample size.

The usage of data from different programmes and regions will improve the sample size and, with the study of the different categories of PCCRC, the surveillance recommendations may even vary, which is why we encourage its use and comparison in the near future owing to the fact that available evidence is still scarce.

## 5. Conclusions

The classification of the WEO experts in interval cancer provides a common framework to monitor and compare the surveillance and results of screening programmes.

Interval type PCCRCs and non-interval type PCCRCs show differences in terms of the characteristics of the lesion detected in the index colonoscopy, its location and morphology. The probability of finding advanced stages in the case of interval type PCCRCs is three times higher than in non-interval type PCCRCs.

The risk of interval type PCCRC is higher in the cases in which low-risk or intermediate-risk adenomas were found, whilst in the case of non-interval type PCCRCs, the risk is higher with high-risk adenomas.

In the non-interval type PCCRCs, no significant differences were found, in relation to the stage of the tumour, between those whose colonoscopy was performed in the recommended time frame (type A) and those whose colonoscopy was delayed (type B).

The proposed classification tool is useful and manageable. Furthermore, it clearly reflects the different entities within the subgroups, thus allowing us to see areas of improvement in the surveillance of the lesions, which will allow us to decrease those cases of interval type PCCRCs with worse prognosis.

## Figures and Tables

**Table 1:** Incidence of post-colonoscopy colorectal cancer (PCCRC) according to the type of lesion detected at index colonoscopy in the 128 interval and non-interval type PCCRCs.

Endoscopic Finding	Interval type PCCRC Incidence 1000 Person-Years (95% CI) (*n* = 50)	Non-Interval Type PCCRCIncidence 1000 Person-Years (95% CI) (*n* = 78)		
		General (*n* = 78)	Non-Interval Type A PCCRC (*n* = 61)	Non-Interval Type B PCCRC (*n* = 17)
All the findings	0.116 (0.088–0.153)	0.160 (0.129–0.200)	0.128 (0.010–0.165)	0.030 (0.019–0.049)
No lesion	0.122 (0.078–0.190)	0.011 (0.003–0.041)	-	0.010 (0.003–0.036)
Inflammatory Bowel Disease	-	0.339 (0.093–1.228)	0.173 (0.030–0.975)	0.147 (0.026–0.828)
Low-Risk Adenoma	0.235 (0.148–0.371)	0.080 (0.039–0.166)	0.047 (0.018–0.121)	0.030 (0.010–0.088)
Intermediate-Risk Adenoma	0.117 (0.065–0.209)	0.094 (0.051–0.173)	0.077 (0.039–0.152)	0.016 (0.004–0.059)
High-Risk Adenoma	0.027 (0.007–0.098)	0.662 (0.510–0.859)	0.580 (0.438–0.768)	0.082 (0.041–0.162)
Inconclusive	-	0.525 (0.093–2.928)	-	0.456 (0.081–2.540)

**Table 2:** Characteristics of PCCRC interval type and PCCRC non-interval type.

Variables	Interval Type PCCRC (*n* = 50)	Non-Interval Type PCCRC (*n* = 78)	OR ^1^	CI	*p*-Value
Sex (*n* (%))					
Men	27 (54)	53 (67.9)	-	-	-
Women	23 (46)	25 (32.1)	-	-	-
Age group (*n* (%))					
*Mean (SD)*	62.78 (5.4)	65.24 (5.05)	-	-	-
50–59 years	13 (26)	10 (12.8)	-	-	-
60–69 years	30 (60)	52 (66.7)	-	-	-
≥70 years	7 (14)	16 (20.5)	-	-	-
Tumour location (*n* (%))					
Caecum	8 (16)	11 (14.1)	-	-	-
Proximal	14 (28)	28 (35.9)	-	-	-
Distal	19 (38)	29 (37.2)	-	-	-
Rectum	9 (18)	10 (12.8)	-	-	-
Tumour stage (*n* (%))					
Early stage (I–II) (Ref.)	27 (54)	61 (78.2)	-	-	-
Advanced stage (III–IV)	23 (46)	17 (21.8)	3.057	1.410–6.625	0.005
Deprivation index (*n* (%))					
Q1 (Very low) (less deprived)	7 (14)	11 (14.1)	-	-	-
Q2 (Low)	6 (12)	15 (19.2)	-	-	-
Q3 (Moderate)	12 (24)	15 (19.2)	-	-	-
Q4 (High)	12 (24)	17 (21.8)	-	-	-
Q5 (Very high) (most deprived)	11 (22)	19 (24.4)	-	-	-
Unknown	2 (4)	1 (1.3)	-	-	-
Time between the index colonoscopy and CRC diagnosis in months [$\bar{x}$, SD, median]	30.92(18.169) 26	35.76 (22.330) 36	-	-	0.314
No lesion	36.74(17.773) 34	52.50 (24.749) 52.5	-	-	0.286
Inflammatory Bowel Disease	-	42.5 (40.305) 42.5	-	-	-
Low-risk adenoma	33.83(20.171) 33.0	64.43 (8.344) 68.0	-	-	0.000
Intermediate-risk adenoma	17.64 (8.310) 18	38.80 (5.029) 37.5	-	-	0.000
High-risk adenoma	22.50 (2.121) 22.5	30.95 (22.418) 20	-	-	0.915

^1^ ORs were only calculated for variables that had a significance *p* < 0.05 in the univariate analysis. Abbreviations: SD, standard deviation.

**Table 3:** Characteristics of the interval type PCCRC and non-interval type PCCRC in terms of the index colonoscopy.

Variables	Interval Type PCCRC (*n* = 50)	Non-Interval Type PCCRC (*n* = 78)	OR ^1^	CI	*p*-Value
Caecal intubation *(n* (%))					
Complete	50 (100)	76 (97.4)	-	-	-
Incomplete	0 (0.0)	1 (1.3)	-	-	-
Non-applicable	0 (0.0)	1 (1.3)	-	-	-
Boston Bowel Preparation Scale (*n* (%))					
Adequate ≥6	41 (82.0)	56 (71.8)	-	-	-
Poor <6	2 (4.0)	10 (12.8)	-	-	-
Non-applicable	7 (14.0)	12 (15.4)	-	-	-
Endoscopic finding (*n* (%)) ^2^					
No lesion	19 (38.0)	2 (2.6)	133.00	22.533–785.013	0.000
Inflammatory Bowel Disease	0 (0.0)	2 (2.6)			
Low-risk adenoma	18 (36.0)	7 (9.0)	72.00	13.707–378.205	0.000
Intermediate-risk adenoma	11 (22.0)	10 (12.8)	30.80	5.915–160.385	0.000
High-risk adenoma (Ref.)	2 (4.0)	56 (71.8)	-	-	-
Inconclusive	0 (0.0)	1 (1.2)	-	-	-
Coincident location (*n* (%)) ^3^					
Yes (Ref.)	12 (24.0)	53 (67.9)	-	-	-
No	38 (76.0)	25 (32.1)	6.713	3.003–15.009	0.000
Location of adenoma resected (*n* (%))					
Caecum	1 (8.3)	5 (9.4)	-	-	-
Proximal colon	5 (41.7)	17 (32.1)	-	-	-
Distal colon	4 (33.3)	27 (50.9)	-	-	-
Rectum	2 (16.7)	4 (7.5)	-	-	-
Paris Classification (*n* (%))					
Is (Ref.)	7 (58.3)	13 (24.5)	-	-	-
Isp	1 (8.3)	5 (9.4)	0.371	0.036–3.838	0.406
Ip	1 (8.3)	21 (39.6)	0.088	0.010–0.803	0.031
IIa/IIb	1 (8.3)	14 (26.4)	0.133	0.014–1.230	0.075
Unknown	2 (16.7)	0 (0.0)	-	-	-
Adenoma retrieved (*n* (%))					
Yes	9 (44.4)	50 (94.3)	-	-	-
No	3 (25.0)	3 (5.7)	-	-	-

^1^ ORs were only calculated for variables that had a significance *p* < 0.05 in the univariate analysis. ^2^ In the calculation of the Odds Ratio, the categories “no lesion” and “inflammatory bowel disease” were unified. ^3^ Location of the CRC coincident with the location of the previously resected adenoma.

**Table 4:** Characteristics of non-interval type A PCCRC and non-interval type B PCCRC.

Variables ^1^	Non-Interval Type A PCCRC (*n* = 61)	Non-Interval Type B PCCRC (*n* = 17)
Sex		
Men	44 (72.1)	9 (52.9)
Women	17 (27.9)	8 (47.1)
Age group (*n* (%))		
Mean (SD)	64.87 (4.836)	66.59 (5.702)
50–59 years	8 (13.1)	2 (11.8)
60–69 years	42 (68.9)	10 (58.8)
≥70 years	11 (18.0)	5 (29.4)
Deprivation index (*n* (%))		
Q1 (Very low)	11 (18.0)	0 (0.0)
Q2 (Low)	11 (18.0)	4 (23.5)
Q3 (Moderate)	12 (19.7)	3 (17.6)
Q4 (High)	12 (19.7)	5 (29.4)
Q5 (Very high)	15 (24.6)	4 (23.5)
Unknown	0 (0.0)	1 (5.9)
Topography (*n* (%))		
Caecum	10 (16.4)	1 (5.9)
roximal	21 (34.4)	7 (41.2)
Distal	23 (37.7)	6 (35.3)
Rectum	7 (11.5)	3 (17.6)
Tumour stage (*n* (%))		
Early stage (I–II)	50 (82.0)	11 (64.7)
Advanced stage (III–IV)	11 (18.0)	6 (35.3)

^1^ ORs were not calculated due to the fact that none of the variables had a significance *p* < 0.05 in the univariate analysis.

**Table 5:** Characteristics of non-interval type A PCCRC and non-interval type B PCCRC in terms of the index colonoscopy.

Variables	Non-Interval Type A PCCRC (*n* = 61)	Non-Interval Type B PCCRC (*n* = 17)	OR ^1^	CI	*p*-Value
Caecal intubation (*n* (%))					
Complete	60 (98.4)	16 (94.1)	-	-	-
Incomplete	0 (0.0)	1 (5.9)	-	-	-
Non-applicable	1 (1.6)	0 (0.0)	-	-	-
Boston Bowel Preparation Scale (*n* (%))					
Adequate ≥6	45 (73.8)	10 (58.8)	13.5	2.368–76.978	0.003
Poor <6 (Ref.)	2 (3.3)	6 (35.3)	-	-	-
Non-applicable	14 (23.0)	1 (5.9)	-	-	-
Endoscopic finding (*n* (%)) ^2^					
No lesion (Ref.)	0 (0.0)	2 (11.8)	-	-	-
Inflammatory Bowel Disease (Ref.)	1 (4.6)	1 (5.9)	-	-	-
Low-risk adenoma	4 (5.1)	3 (17.6)	4.00	0.265–60.325	0.317
Intermediate-risk adenoma	8 (13.1)	2 (11.8)	12.00	0.773–186.362	0.076
High-risk adenoma	48 (78.7)	8 (47.1)	18.00	1.660–195.215	0.017
Inconclusive	0 (0.0)	1 (5.9)	-	-	-
Coincident location (*n* (%)) ^3^					
Yes	45 (73.8)	8 (47.1)	3.164	1.043–9.602	0.042
No (Ref.)	16 (26.2)	9 (52.9)	-	-	-
Location of adenoma resected (*n* (%))					
Caecum	1 (5.9)	10 (16.4)	-	-	-
Proximal colon	7 (41.2)	21 (34.4)	-	-	-
Distal colon	6 (35.3)	23 (37.7)	-	-	-
Rectum	3 (17.6)	7 (11.5)	-	-	-
Paris Classification (*n* (%))					
Is (Ref.)	12 (19.7)	1 (5.9)	-	-	-
Isp	2 (3.3)	3 (17.6)	0.056	0.004–0.838	0.037
Ip	20 (32.8)	1 (5.9)	1.667	0.095–29.182	0.727
IIa/IIb	11 (18.0)	3 (17.6)	0.306	0.028–3.390	0.334
CRC no coincident with the previously resected	16 (26.2)	9 (52.9)	-	-	-
Adenoma retrieved (*n* (%))					
Yes	42 (68.9)	8 (47.1)	-	-	-
No	4 (6.6)	0 (0.0)	-	-	-
Not applicable	15 (19.2)	9 (11.5)	-	-	-

^1^ ORs were only calculated for variables that had a significance *p* < 0.05 in the univariate analysis. ^2^ In the calculation of the Odds Ratio, the categories “no lesion” and “inflammatory bowel disease” were unified. ^3^ Location of the CRC coincident with the location of the previously resected adenoma.

## Data Availability

The data presented in this study are available on request from the corresponding author. The data are not publicly available due to their containing information that could compromise the privacy of research participants.
